# 3D‐Printed Laminae for Kyphosis in Ankylosing Spondylitis During Pedicle Subtraction Osteotomy

**DOI:** 10.1111/os.70074

**Published:** 2025-05-22

**Authors:** Yilin Lu, Gao Si, Mingxiao Bai, Yongqiang Wang, Yun Tian, Weishi Li, Miao Yu, Yu Wang

**Affiliations:** ^1^ Department of Orthopaedics Peking University Third Hospital Beijing China; ^2^ Engineering Research Center of Bone and Joint Precision Medicine Beijing China; ^3^ Beijing Key Laboratory of Spinal Disease Research Beijing China; ^4^ Department of Orthopaedics Rizhao Hospital of Traditional Chinese Medicine Rizhao China; ^5^ Department of Orthopaedics Peking University First Hospital Beijing China

**Keywords:** 3D‐printed, ankylosing spondylitis, kyphosis, pedicle subtraction osteotomy

## Abstract

**Objective:**

Ankylosing spondylitis (AS) often presents with spinal kyphosis, and pedicle subtraction osteotomy (PSO) is a common surgical technique for correcting AS‐related kyphosis. However, after PSO, the posterior column lacks rigid bone support, potentially leading to intervertebral disc mobility and loss of correction. This study aims to introduce a novel 3D‐printed laminae for the treatment of AS‐related kyphosis.

**Methods:**

This is a retrospective cohort study. A total of 48 patients receiving posterior correction surgeries between December 2021 and January 2022 were included and divided into two groups according to whether they accepted the 3D‐printed laminae. We propose a novel approach using 3D‐printed laminae to enhance posterior column stability and reduce deformity loss. Sixteen patients receiving 3D‐printed laminae and 32 patients who did not receive that device. We collected preoperative and postoperative radiographic parameters, perioperative data, and patient‐reported clinical scores. Statistical analysis involved independent sample *t* tests or randomization tests for continuous variables and chi‐square tests for categorical variables.

**Results:**

In the implanted group, kyphosis was corrected from 75.88° preoperatively to 27.06° postoperatively, and in the unimplanted group, from 70.98° to 28.42°. At the last follow‐up, the ΔGK (global kyphosis) was 1.76° in the implanted group and 2.50° in the unimplanted group. PJA was 9.77° in the implanted group and 15.45° in the unimplanted group, showing significant differences. Two patients in the unimplanted group experienced sagittal reconstruction failure. Health‐related quality of life (HRQoL) scores improved in the implanted group, with back pain scores of 2.63 and Oswestry Disability Index (ODI) scores of 13.50.

**Conclusions:**

Our study introduces a novel 3D‐printed laminae technique for AS‐related kyphosis, aiding in maintaining sagittal balance. Patients reported improved subjective outcomes, including reduced pain and better HRQoL.

## Graphical abstract

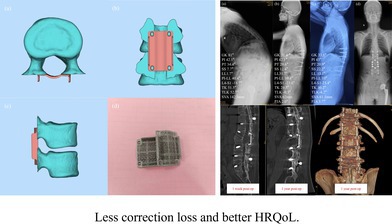

## 1 Introduction

Ankylosing spondylitis (AS) is a chronic systemic immune and inflammatory disease characterized by chronic inflammation affecting the axial skeleton, including the sacroiliac joints and hip joints. Spinal arthritis is one subtype of AS, presenting with lesions in the attachment of ligaments, joint fibers, or bone stiffness, restricted hip joint movement, spinal stiffness, and spinal kyphosis [1]. The prevalence of AS‐related kyphosis is approximately 2% [2]. Severe spinal kyphosis significantly impacts mobility, pulmonary function, and quality of life. Additionally, the high cost of surgical treatment and mechanical complications pose challenges to patients' economic well‐being and overall quality of life [3, 4].

Pedicle subtraction osteotomy (PSO) is a commonly used procedure for correcting kyphosis in AS patients [5]. It effectively restores sagittal alignment, allowing most patients to regain the ability to walk with horizontal vision and lie on their backs after surgery [6]. However, traditional PSO surgery lacks rigid bone support in the posterior spine, relying solely on anterior vertebral bodies for stability. At the same time, the rear screws and connecting rods bear the stress alone, without other structures sharing the load with them. This raises concerns about loss of correction and implant failure. Factors such as spinal mobility contributing to nonfusion of bone grafts and heterotopic ossification may result in sagittal decompensation during follow‐up, further progressing to sagittal reconstruction failure [7, 8]. Therefore, optimizing post‐PSO spinal stability and enhancing bone fusion rates remain critical topics of debate among orthopedic surgeons.

3D‐printed implants exhibit excellent biocompatibility, corrosion resistance, and mechanical strength. Their porous structure also facilitates bone fusion [9]. In recent years, 3D printing technology has been widely used for creating interbody fusion devices, vertebral bodies, and screws [9, 10]. In our study, we developed personalized 3D‐printed laminae to (i) address the anatomical deficiencies in the postoperative spine and (ii) create an optimal environment for bone fusion, resulting in (iii) improved fusion rates and better sagittal alignment.

## 2 Materials and Methods

### 2.1 Study Design

This is a retrospective analysis of consecutively treated AS patients undergoing PSO surgeries at the same center. Included patients had a minimum follow‐up period of 2 years. Following approval by the Institutional Review Board (IRB number: M20250179), data from spinal deformity surgeries conducted at the same center between December 2021 and January 2022 were collected.

### 2.2 Patients

Patient inclusion criteria comprised the following: (1) availability of complete preoperative, postoperative, and last‐follow‐up radiological data, along with patient‐reported outcome score data; (2) diagnosis of AS; preoperative TK > 50° and a diagnosis of kyphosis; (3) undergoing PSO surgery; and (4) age within the range of 18–80 years. Exclusion criteria included: (1) patients with incomplete or nonfull‐spine X‐ray imaging data, as well as those with incomplete patient‐reported outcomes; (2) lumbar scoliosis secondary to trauma, neurodegenerative diseases (such as Parkinson's disease), and other conditions; (3) concomitant with other spine disorders such as Scheuermann's disease and degenerative spinal kyphosis; (4) history of spinal surgery. Patients were classified according to the 301 classification [11]. Using propensity score matching, patients who received 3D‐printed laminae implants were included in the implanted group, while those without implants constituted the unimplanted group, maintaining a 1:2 ratio. Variables included in the propensity score included age, BMI, presence of posterior internal fixation, smoking status, and length of hospital stay. For the possible postoperative complications of 3D printed implants, we have fully explained to the patients, and the patients would decide whether to accept this treatment and sign the informed consent.

### 2.3 3D‐Printed Laminae

**FIGURE 1:**
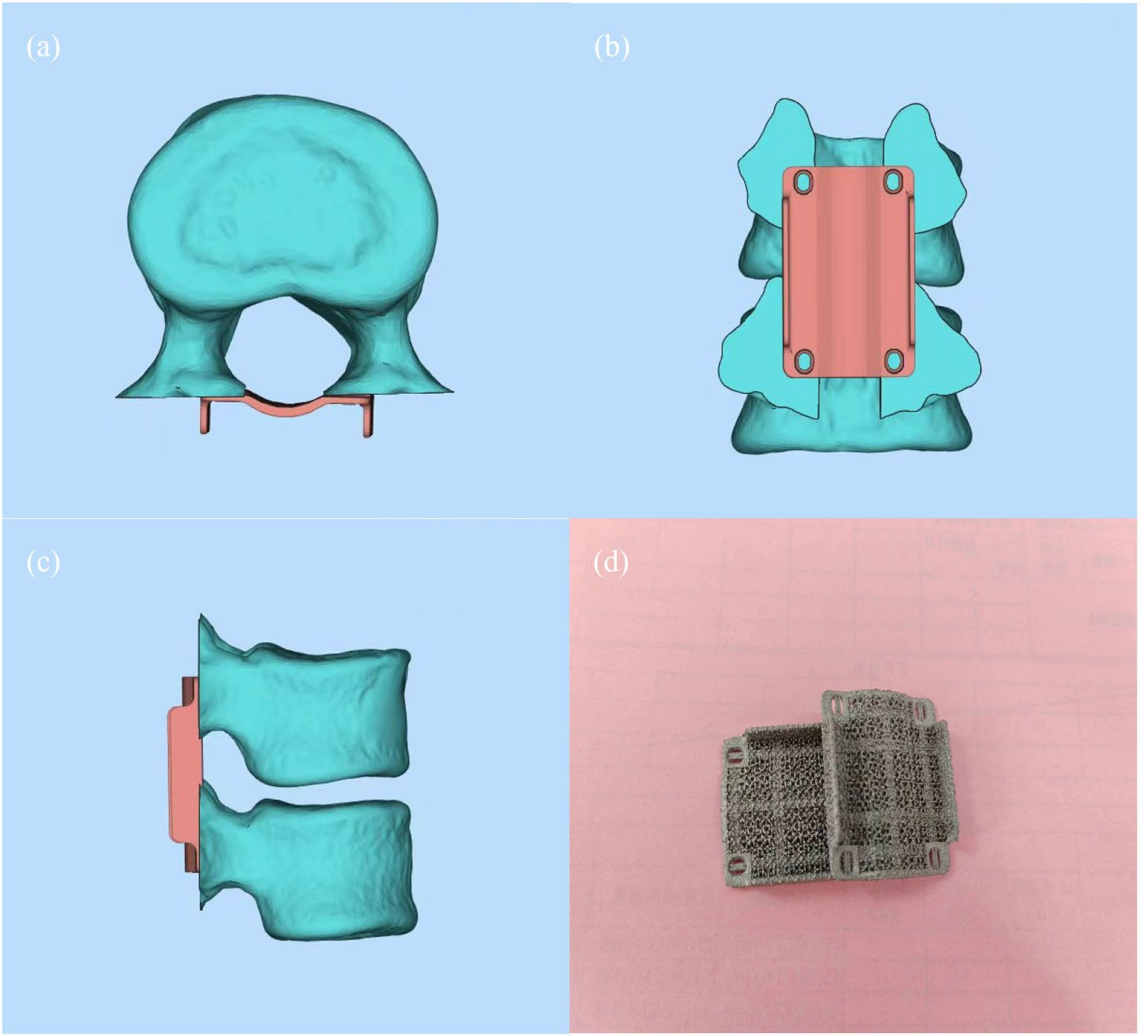
3D‐printed laminae. (a)–(c) show the vertebral body and 3D‐printed laminae in the modeling software. (d) is a physical picture.

The patient imaging data in DCM format was imported into Mimics 21.0 (Materialize Corp., Leuven, Belgium) for modeling, and then the models were imported into Unigraphics NX 12.0 (Siemens PLM Software, Plano, Texas, US) to design a customized 3D‐Printed Laminae. After the design was completed, the finished products were printed (Figure 1). The devices were manufactured by Beijing AK Medical Co. Ltd.

### 2.4 Surgical Procedure

The skin and subcutaneous tissue were incised sequentially, then subperiosteally detached; the paraspinal muscles were subperiosteally detached to the outer edge of the articular processes. Twelve (or 14) screws were inserted into the pedicles of T11, T12, L1, L3, and L5 (in some patients also T10). Bilateral PSO was performed at L2, interbody cages were placed, short working rods were inserted bilaterally, and the abdominal pads were removed. Alternating compression was applied until the vertebral bodies and bilateral lamina osteotomy sites were tightly closed. The working rods were then replaced with long rods, and all nuts were tightened. A 3D‐printed lamina was then placed and fixed with four short screws. A bone graft bed was prepared on the surface of the bilateral lamina using a small bone chisel. Interlaminar bone grafting was performed using excised bone fragments; meticulous hemostasis was achieved, and one drainage tube was placed. After confirming the correct count of instruments and gauze, the lumbar fascia, subcutaneous tissue, and skin were sutured sequentially, and sterile dressings were applied for fixation.

### 2.5 Data Collection and Statistical Analysis

Demographic data collected included age at surgery, gender, height, weight, BMI, underlying conditions, and follow‐up duration. Radiological information collected included whole‐spine X‐ray data preoperatively, 5–7 days postoperatively, and at the last follow‐up, encompassing coronal Cobb angle, global kyphosis (GK), pelvic incidence (PI), pelvic tilt (PT), sacral slope (SS), lumbar lordosis (LL), thoracic kyphosis (TK), thoracolumbar kyphosis (TLK), PI‐LL mismatch, L4‐S1 lordosis, sagittal vertical axis (SVA), and proximal junctional angle (PJA), as well as bone graft fusion status. Parameters were measured using Surgimap 2.3.2.1 (Nemaris Inc., NY, US). Continuous bone trabeculae observed on CT are considered successful fusion. All radiological data were measured by two experts with over 5 years of work experience. Usually, the measurement is done separately by two attending physicians. The results presented in the paper are the average of the two doctors' measurements. However, if the results measured by two attending physicians are significantly different, we will ask a doctor with an associate senior title or above to measure and rule on the results. These doctors had more than 5 years of experience and treated at least two patients per week with spinal deformities. These three researchers were solely responsible for data measurement and were blinded to the study design. Additionally, surgical details were recorded, including operative time, intraoperative blood loss, intraoperative transfusion volume, and bone grafting. Patient‐reported outcome scores were collected, including visual analogue scale (VAS), Oswestry Disability Index (ODI) scores, and the Japanese Orthopedic Association (JOA) scores. Before and after surgery, patients were asked to fill out paper questionnaires. During the follow‐up period, we collected patient scores by means of an online questionnaire. The patients underwent imaging examinations at the re‐examination outpatient department and filled in the subjective score questionnaires.

Statistical analysis was performed using the IBM SPSS Statistics 27.0 (IBM Corp., Armonk, NY, USA), with a significance level set at *p* < 0.05. Continuous variables following a normal distribution were expressed as mean ± standard deviation. The *t* test was used for comparisons between two groups, while one‐way ANOVA was used for comparisons among multiple groups. The chi‐square test was used for comparisons of multiple components. Categorical variables were expressed as frequencies, and comparisons between groups were made using the chi‐square test.

## 3 Results

### 3.1 Baseline Data and Surgical Details

**FIGURE 2:**
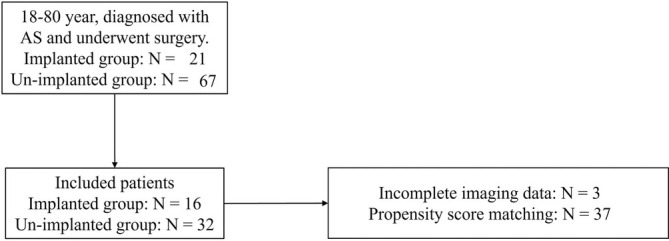
Flow diagram.

A total of 48 patients were included in the study, of which 24 (50%) were male (Figure 2). The implanted group included 16 patients, while the unimplanted group included 32 patients. The average age of all patients was 33.63 years, and the average number of fused segments was 6.10 (Table 1). The operative time and intraoperative blood transfusion volume were approximately equal between the two groups, but there was a significant difference in intraoperative blood loss, with the implanted group and unimplanted group having 1475.00 and 1118.75 mL, respectively. Additionally, there was no significant difference in bone grafting between the two groups (Table 2). Overall, the surgical outcomes were satisfactory, with a significant correction of kyphosis and better sagittal balance, and sagittal parameters maintained at the last follow‐up. The overall fusion rate of interlaminar bone grafts was 81.25% (Table 3).

**TABLE 1:** Demographic data of patients.

Group	Total	Planted group	Unplanted group	*p*
*N*	48	16	32	
Age (years)	33.63 ± 4.47	33.56 ± 4.66	33.66 ± 4.45	0.947
BMI (kg/m^2^)	23.92 ± 4.18	22.96 ± 3.30	24.40 ± 4.53	0.266
Male (%)	24 (50)	8 (50)	16 (50)	1.000
301 classification (%)				0.629
I	3 (6.25)	1 (6.25)	2 (6.25)	
IIA−	1 (2.08)	0	1 (3.13)	
IIA+	14 (29.17)	5 (31.25)	9 (28.13)	
IIB−	1 (2.08)	0	1 (3.13)	
IIB+	17 (35.42)	7 (43.75)	10 (31.25)	
IIIA−	4 (8.33)	0	4	
IIIA+	6 (12.50)	3 (18.75)	3 (9.38)	
IIIB−	0	0	0	
IIIB+	2 (4.17)	0	2 (6.25)	
IV	0	0	0	
Follow‐up duration/m	24.54 ± 0.92	24.69 ± 0.95	24.47 ± 0.915	0.444
Hospital stay/d	21.31 ± 6.73	23.81 ± 7.34	20.06 ± 6.14	0.068
Cost/CNY	116,420.68 ± 3271.31	116,302.22 ± 3366.72	116,481.49 ± 3232.55	0.678
Fused segments	6.10 ± 0.59	6.19 ± 0.40	6.06 ± 0.67	0.496
Interbody fusion (%)	41 (85.42%)	13 (81.25)	28 (87.50)	0.261
Basic situation (%)				
Hypertension	2 (4.17)	2 (12.50)	0	0.261
Diabetes	0	0	0	0.135
CHD	0	0	0	0.135
Smoke	3 (6.25)	1 (6.25)	2 (6.25)	0.063
PI	19 (39.58)	6 (37.5)	13 (40.63)	0.136

Abbreviations: BMI: body mass index; CHD: coronary heart disease; PI: pulmonary insufficiency.

**TABLE 2:** Perioperative presentation of patients.

	Total	Implanted group	Unimplanted group	*p*
OD	359.19 ± 49.65	372.38 ± 27.40	352.59 ± 56.92	0.196
IBL	1237.50 ± 482.73	1475.00 ± 418.73	1118.75 ± 474.13	0.014*
IBTV	583.33 ± 390.08	665.63 ± 373.60	542.19 ± 397.39	0.306
HB at 2 days	102.81 ± 15.01	100.94 ± 14.51	103.75 ± 15.40	0.546
Bone grafting (%)				0.117
Intervertebral	41 (85.42)	13 (81.25)	28 (87.50)	
Paravertebral	14 (29.17)	7 (43.75)	7 (21.88)	
Intertransverse	5 (10.42)	0	5 (15.63)	

Abbreviations: HB: hemoglobin; IBL: intraoperative blood loss; IBTV: intraoperative blood transfusion volume; OD: operation duration.

**TABLE 3:** Sagittal parameters of all patients: Preoperatively, postoperatively, and the last follow‐up.

	Pre‐op	Post‐op	Last follow‐up (LFU)	*p* (pre‐op vs. post‐op)	*p* (Post‐op vs. LFU)	*p* (Pre‐op vs. LFU)
Cobb Angle	31.99 ± 14.43	13.16 ± 9.39	12.89 ± 8.82	0.000	0.597	0.000
PI	46.93 ± 13.10	47.15 ± 13.16	47.29 ± 13.35	0.994	0.291	0.334
PT	25.65 ± 11.49	18.89 ± 10.86	23.49 ± 10.76	0.000	0.026	0.000
SS	21.25 ± 13.27	28.51 ± 10.19	24.50 ± 11.39	0.000	0.000	0.001
LL	20.48 ± 24.42	34.79 ± 15.96	29.64 ± 16.84	0.000	0.000	0.000
PI‐LL	23.53 ± 2.03	16.12 ± 1.39	18.19 ± 1.68	0.000	0.000	0.000
L4‐S1	31.27 ± 17.91	26.16 ± 14.77	25.73 ± 12.78	0.051	0.785	0.060
GK	31.67 ± 14.62	20.41 ± 11.79	27.94 ± 14.24	0.000	0.000	0.003
TK	18.28 ± 16.55	20.79 ± 10.58	23.84 ± 12.80	0.015	0.000	0.000
SVA	51.13 ± 49.76	24.89 ± 42.58	47.25 ± 42.21	0.000	0.000	0.462
PJA		6.27 ± 6.19	8.20 ± 8.06		0.002	

Abbreviations: GK: global kyphosis, LL: lumbar lordosis, PI: pelvic incidence, PJA: proximal junctional angle, PT: pelvic tilt, SS: sacral slope, SVA: sagittal vertical axis, TK: thoracic kyphosis, TLK: thoracolumbar kyphosis.

### 3.2 Imaging Data and Subjective Scores

At 1 week postoperatively, there were no significant differences in sagittal parameters and surgery outcomes between the two groups. The kyphosis in the implanted group and unimplanted group was corrected from 75.88° and 70.98° to 27.06° and 28.42°, respectively. However, significant differences in ΔGK (1.76° vs. 2.50°) and PJA (9.77° vs. 15.45°) were observed at the last follow‐up. Two patients in the unimplanted group were identified as having sagittal reconstruction failure (Table 4). The ICC values of all imaging data were > 0.9, demonstrating good consistency. Regarding HRQoL (Table 5), patients reported significantly better scores postoperatively and at follow‐up compared to preoperatively. Preoperative VAS (Back), VAS (Leg), and JOA scores were 6.33, 4.15, and 13.04, respectively, while postoperative scores were 3.12, 3.38, and 16.90, respectively. At follow‐up, these scores were 4.02, 3.60, and 17.21, respectively. There were no significant differences in postoperative scores between the two groups. At follow‐up, patients in the implanted group showed considerable improvement in back pain and ODI scores compared to the other group, with back pain scores of 2.63 and 4.38, and ODI scores of 13.50 and 19.34, respectively. Finally, there was no significant difference in perioperative and long‐term complication rates between the two groups (Table 6).

**TABLE 4:** Sagittal parameters at different groups: implanted group (*n* = 16) and unimplanted group (*n* = 32).

		Implanted group	Unimplanted group	*p*
Pre‐op	Cobb angle	7.98 ± 6.34	9.21 ± 5.60	0.499
	PI	47.53 ± 14.11	46.05 ± 12.32	0.459
	PT	42.67 ± 17.34	14.42 ± 18.50	0.824
	SS	3.26 ± 13.16	−1.86 ± 15.87	0.274
	LL	1.03 ± 20.09	0.28 ± 18.55	0.900
	PI‐LL	45.01 ± 24.27	40.65 ± 20.52	0.520
	L4‐S1	−23.38 ± 12.17	−16.00 ± 15.36	0.102
	GK	75.88 ± 19.67	70.98 ± 22.24	0.462
	TK	39.84 ± 12.82	48.67 ± 18.16	0.090
	TLK	31.79 ± 14.21	28.26 ± 12.06	0.375
	SVA	133.75 ± 66.73	152.72 ± 49.64	0.276
Post‐op	Cobb angle	4.83 ± 3.10	6.66 ± 5.97	0.257
	PI	43.66 ± 14.65	40.11 ± 14.01	0.350
	PT	22.24 ± 14.24	22.05 ± 10.09	0.958
	SS	21.39 ± 11.94	20.04 ± 8.83	0.662
	LL	39.06 ± 22.43	40.09 ± 12.71	0.840
	PI‐LL	2.20 ± 25.38	−0.216 ± 12.26	0.657
	L4‐S1	−21.41 ± 11.73	−17.83 ± 14.13	0.387
	GK	27.06 ± 15.83	28.42 ± 16.67	0.788
	TK	41.28 ± 13.20	47.01 ± 17.88	0.263
	TLK	−9.20 ± 12.03	−5.89 ± 17.42	0.499
	SVA	60.41 ± 38.53	72.56 ± 37.25	0.298
	PJA	10.81 ± 5.89	12.94 ± 7.87	0.345
LFU	Cobb angle	4.19 ± 3.09	6.05 ± 6.50	0.323
	PI	48.79 ± 18.39	40.57 ± 6.35	0.094
	PT	29.70 ± 15.05	20.88 ± 9.25	0.086
	SS	19.19 ± 13.52	19.68 ± 10.42	0.917
	LL	36.55 ± 17.84	40.42 ± 16.61	0.564
	PI‐LL	11.23 ± 8.75	0.18 ± 14.53	0.041*
	L4‐S1	−18.27 ± 12.72	−27.62 ± 12.49	0.098
	GK	28.82 ± 15.69	30.92 ± 16.68	0.678
	ΔGK	1.76 ± 1.39	2.50 ± 0.76	0.022*
	TK	43.50 ± 14.15	54.06 ± 12.06	0.134
	TLK	−3.75 ± 19.03	−4.10 ± 14.99	0.966
	SVA	55.55 ± 6.41	55.32 ± 19.53	0.982
	PJA	9.77 ± 5.24	15.45 ± 6.50	0.028
	Fusion rate (%)	14 (87.5%)	25 (78.13%)	0.695
*	SRF (%)	0	2 (6.25%)	0.798

Abbreviation: SRF: sagittal reconstruction failure.

* *p*﹤0.05.

**TABLE 5:** Patient‐reported clinical scores.

		Total	Implanted group	Unimplanted group	*p*
Pre‐op	VAS (Back)	6.33 ± 1.75	6.63 ± 1.86	6.19 ± 1.71	0.421
	VAS (Leg)	4.15 ± 2.93	4.06 ± 2.98	4.19 ± 2.96	0.891
	ODI	28.25 ± 7.66	28.94 ± 6.77	27.91 ± 8.15	0.665
	JOA	13.04 ± 5.63	14.31 ± 6.62	12.41 ± 5.06	0.273
Post‐op	VAS (Back)	3.12 ± 2.15^b^	3.13 ± 2.60^b^	3.13 ± 1.93^b^	1.000
	VAS (Leg)	3.38 ± 1.85	3.63 ± 1.67	3.26 ± 1.95	0.514
	ODI	24.46 ± 8.42^a^	25.50 ± 6.09	23.94 ± 9.42	0.550
	JOA	16.90 ± 3.70^b^	16.88 ± 3.28	16.91 ± 3.94^b^	0.978
LFU	VAS (Back)	3.79 ± 1.91^b^	2.63 ± 1.59^b^	4.38 ± 1.81^b^	0.002**
	VAS (Leg)	3.60 ± 1.67	3.06 ± 1.18	3.88 ± 1.83	0.113
	ODI	17.40 ± 7.83^b^	13.50 ± 6.22^a^	19.34 ± 7.91^a^	0.013**
	JOA	17.21 ± 3.82^b^	18.44 ± 4.20^a^	16.59 ± 3.52^b^	0.115

Abbreviation: LFU: last follow‐up.

^a^ Compared with preoperation, *p* < 0.05.

^b^ Compared with preoperation, *p* < 0.01.

** *p*﹤0.01.

**TABLE 6:** Complications between implanted group and unimplanted group.

	Implanted group	Unimplanted group	*p*
Perioperative complication(%)			
Waist/leg weakness/numbness	2 (12.50)	6 (18.8)	0.701
Reoperation	0 (0)	0 (0)	/
Cerebrospinal fluid leak	1 (6.25)	1 (3.13)	0.610
Wound infection	1 (6.25)	3 (9.38)	0.712
Hematoma	0 (0)	1 (3.13)	0.475
Pulmonary embolism	0 (0)	0 (0)	/
Gastrointestinal symptoms	2 (12.50)	5 (15.63)	0.772
Late complication(%)			
PJK	2 (12.50)	7 (21.88)	0.433
DJK	0	1 (3.13)	0.475
Screw Loosening	0 (0)	0 (0)	/
Rod Breakage	0 (0)	1 (3.13)	0.475
Cage Subsidence	2 (12.5)	5 (15.63)	0.772
Pseudarthrosis	1 (6.25)	4 (12.50)	0.504

### 3.3 Representative Cases

**FIGURE 3:**
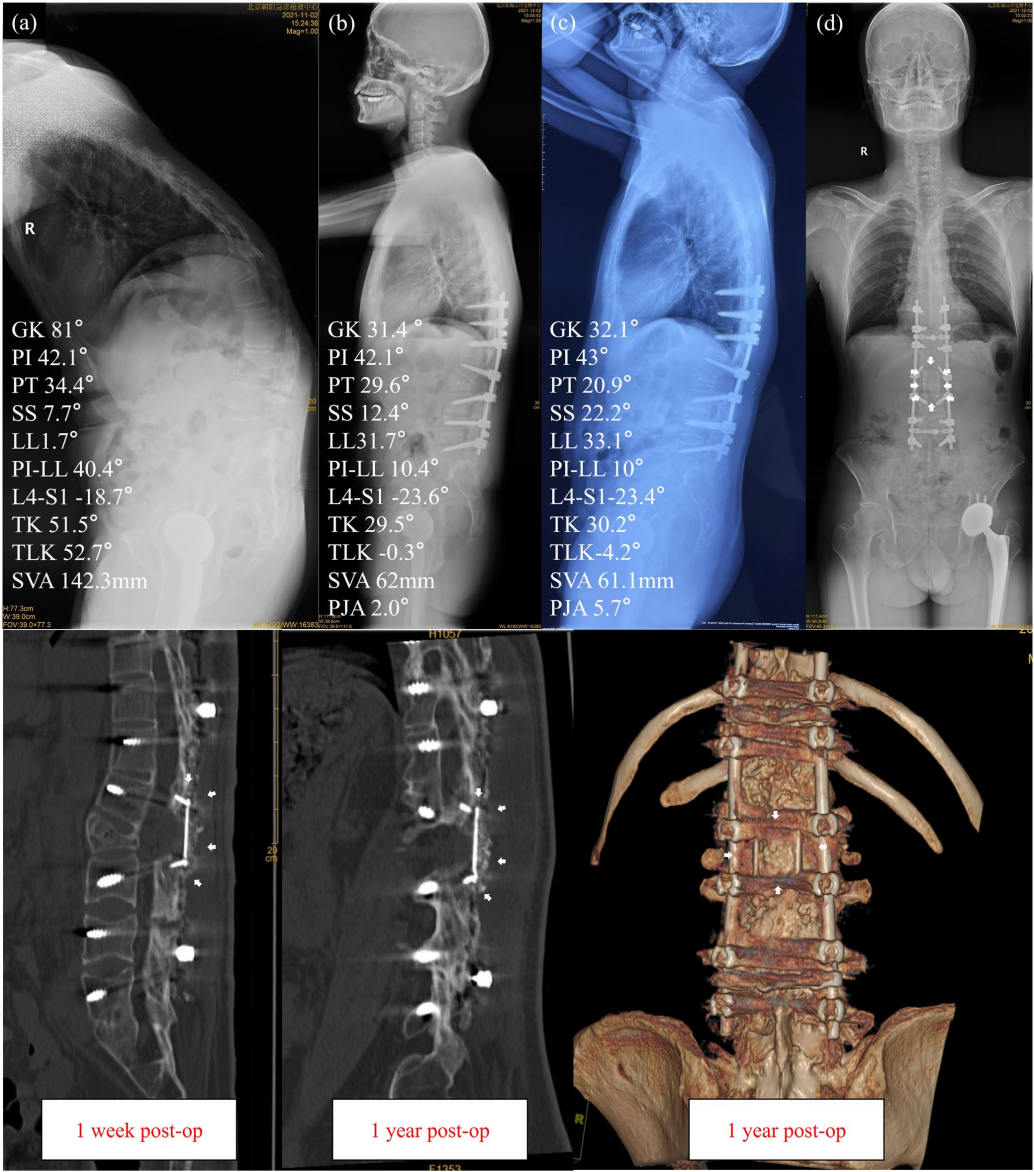
A patient implanted with a 3D‐Printed Laminae. (a)–(c) presents information about the patient's sagittal position before, after surgery, and at follow‐up; (d) Imaging findings of 3D printed laminae were shown; (e)–(g) showed bone graft fusion at 1 week after surgery and at follow‐up. At 1 year after surgery, there was significant bone growth in the implant.

Figure 3 shows a patient who received a 3D‐printed lamina implant. The patient achieved good sagittal balance after surgery, and there was no significant loss of correction at the last follow‐up. Clinical scores reported by the patient did not worsen, and there were no mechanical complications such as PJK or rod breakage. In Figure 3d, the white arrow indicates the morphology and position of the 3D‐printed lamina.

**FIGURE 4:**
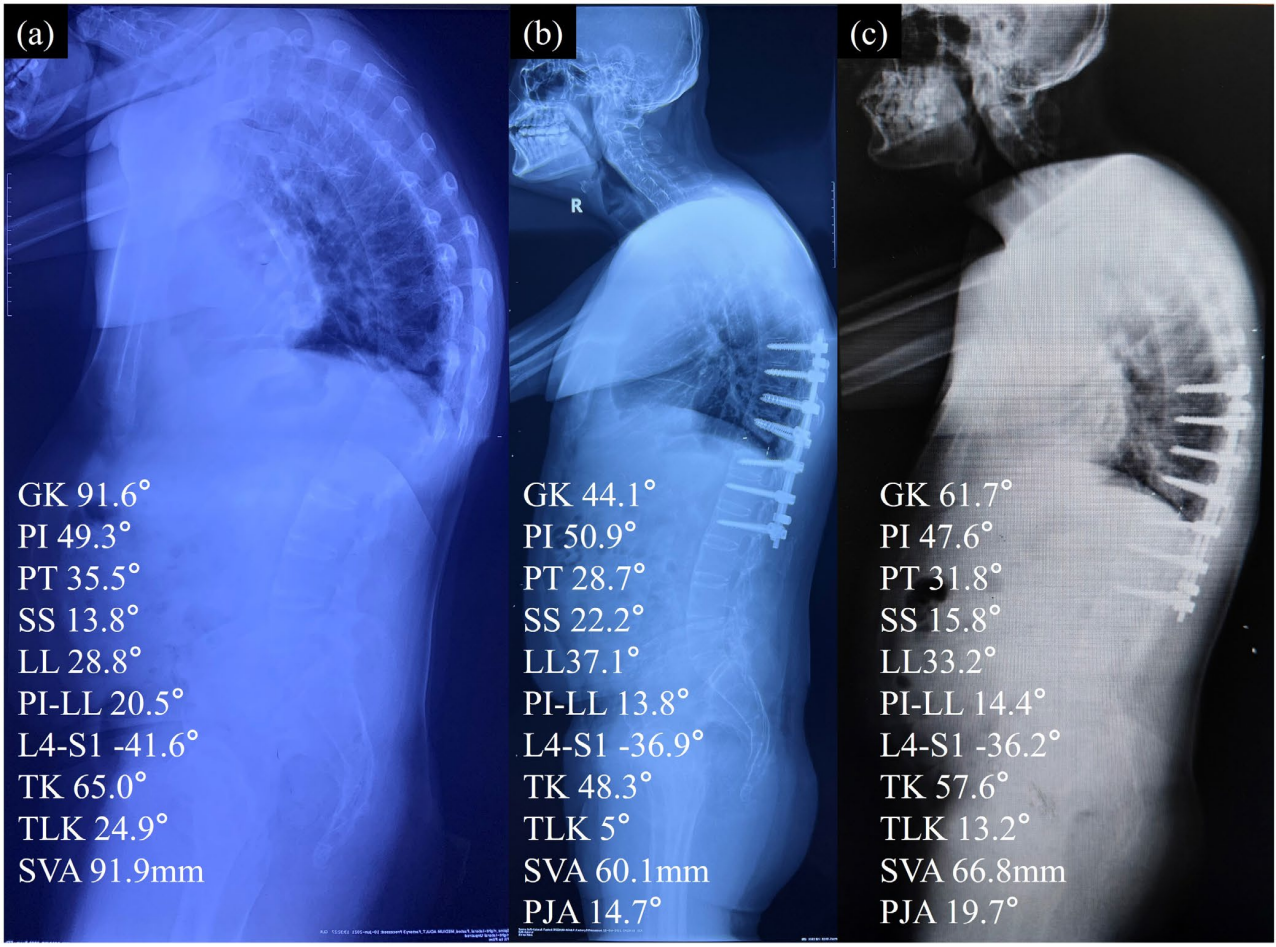
A patient without 3D‐printed laminae. The postoperative kyphosis of the patient was: GK 44.1°, TK 48.3°, TLK 5°. During follow‐up, the above parameters were lost, which were 61.7°, 57.6°, 13.2°, respectively.

Figure 4 displays a patient who did not receive a 3D‐printed lamina implant. This patient experienced varying degrees of rebound in GK, TK, and TLK at the last follow‐up, leading us to consider this case as a failed correction.

## 4 Discussion

The main causes of kyphosis include congenital factors [12], degenerative changes [13], vertebral infections (such as spinal tuberculosis) [14], AS [15], and Scheuermann's disease [16]. Among these, higher complexity and specificity in kyphosis caused by AS make the improvement of surgical outcomes a focal point of research. Surgical correction methods include PSO, Smith‐Petersen osteotomy (SPO), vertebral column resection (VCR), and so on, but the choice of surgical technique remains inconclusive [17]. This study introduced a novel 3D‐printed lamina in order to help maintain the sagittal alignment and reduce postoperative complications.

### 4.1 Surgical Concept

PSO, as a highly invasive surgery, has always been a focus of scholars concerning its efficacy and safety. Studies have reported that post‐PSO GK loss ranges between 2.31° and 2.82°, and this loss increases with longer follow‐up periods [5, 18, 19]. Traditional PSO surgery leads to two structural deficiencies in the spine. First is the complete loss of posterior structures in the osteotomy region, making this area fully supported by the anterior and middle columns of the spine in the vertical direction, leading to a tendency for corrective rebound. Second, osteotomy causes excessive expansion of the intervertebral disc space, allowing for disc mobility, which is one of the reasons for loss of correction, and this effect exacerbates with the progression of disc degeneration. The relative motion of different parts of the spine becomes a disadvantage for achieving satisfactory bone graft fusion [7, 20]. Additionally, during the intraoperative separation of nerve roots or adhesions between the dura mater and surrounding tissues, the dural sac may be torn. The application of 3D printing technology in spinal surgery has been a hot topic in recent years, but previous 3D printing has mostly been used for creating vertebral bodies, titanium cages, and so on, or for surgical planning [21, 22]. Therefore, we aim to achieve our goal from a new perspective—by strengthening the posterior column to reduce loss of correction, protect the dural sac, and reduce internal fixation failure.

Patients implanted with 3D‐printed laminae had more intraoperative blood loss, which may be related to the longer surgery time required for fixing this device (although not statistically different) and the additional surgical procedures leading to bleeding. However, blood routine measurements taken 2 days postoperatively showed no significant difference in HB between the two groups. Therefore, we believe that despite the higher intraoperative blood loss, this issue can be compensated by the patient's own compensation mechanisms and reasonable perioperative fluid and nutritional management.

### 4.2 Imaging Outcomes

The 3D‐printed laminae provide a broad bone grafting surface, allowing for sufficient bone grafting on the lamina surface and the original spinous process area. On the other hand, titanium alloy, the commonly used material for 3D printing, with its microporous structure and interconnected channels between pores, creates an ideal environment for bone fusion [23]. Additionally, the custom‐made implants tailored to the patient's lamina width fit the patient's anatomical characteristics, reducing the possibility of postoperative implant failure [24]. Therefore, as shown in Figure 2, the CT scan at 1 year postoperatively shows significant bone growth around the 3D‐printed laminae, contributing significantly to the maintenance of sagittal balance. The immediate postoperative outcomes were similar between the two groups. However, at the last follow‐up, there were significant differences in ΔGK and PJA, further demonstrating the stabilizing effect of bone growth around the 3D‐printed laminae. The excellent fusion rate and absence of mechanical complications in the implanted group also confirm the performance of this device.

At the last follow‐up, the VAS back pain scores and ODI scores of patients in the implanted group were significantly better than those of the other group. This is partly due to the good maintenance of sagittal parameters and partly due to the reduced irritation to nerves provided by the personalized customization of the device.

### 4.3 Strengthens and Limitations

This study introduced a novel 3D‐printed laminae which helps bone fusion and maintains the stability of the spine. However, there are some limitations. First, this is a single‐center study, lacking external validation. Also, the relatively small patient population may increase the errors.

### 4.4 Prospects of Clinical Application

In the future, we will further increase the sample size to obtain more accurate results. It is known that serious kyphosis can be observed not only in AS patients, but also in diseases such as AIS and tuberculosis. Thus, we will try to expand the application conditions to evaluate its effect in all kinds of kyphosis. Considering that four small screws were placed when securing the device, which may contribute to providing immediate postoperative stability, biomechanical experiments will also be conducted to verify its effects.

## 5 Conclusion

This study proposes a novel 3D‐printed lamina technique for spinal kyphosis secondary to AS. At the last follow‐up, patients maintained good global sagittal balance, and their subjective experiences, including pain symptoms and quality of life scores, showed significant improvement.

## Author Contributions

Yilin Lu collected the data and wrote the original manuscript. Gao Si performed the data analysis, reviewed the manuscript, and made the figures and tables. Mingxiao Bai assisted in the surgeries. Yongqiang Wang and Yun Tian participated in data collection and analysis. Weishi Li, Miao Yu, and Yu Wang discussed and suggested the idea of the study, reviewed the original manuscript. Miao Yu and Yu Wang agreed on the final version of the manuscript.

## Conflicts of Interest

The authors declare no conflicts of interest.
